# A Summer Mortality Outbreak of Lactococcosis by *Lactococcus garvieae* in a Raceway System Affecting Farmed Rainbow Trout (*Oncorhynchus mykiss*) and Brook Trout (*Salvelinus fontinalis*)

**DOI:** 10.3390/ani9121043

**Published:** 2019-11-29

**Authors:** Paolo Pastorino, Ana Isabel Vela Alonso, Silvia Colussi, Giulia Cavazza, Vasco Menconi, Davide Mugetti, Marzia Righetti, Raffaella Barbero, Gaetano Zuccaro, José Francisco Fernández-Garayzábal, Alessandro Dondo, Pier Luigi Acutis, Marino Prearo

**Affiliations:** 1 Istituto Zooprofilattico Sperimentale del Piemonte, Liguria e Valle d’Aosta, via Bologna 148, 10154 Torino, Italy; silvia.colussi@izsto.it (S.C.); g.cavazza90@hotmail.it (G.C.); vasco.menconi@izsto.it (V.M.); davide.mugetti@izsto.it (D.M.); marzia.righetti@gmail.com (M.R.); gaetanobem@hotmail.it (G.Z.); alessandro.dondo@izsto.it (A.D.); pierluigi.acutis@izsto.it (P.L.A.); marino.prearo@izsto.it (M.P.); 2 Centro de Vigilancia Sanitaria Veterinaria (VISAVET), Universidad Complutense de Madrid, Avenida Puerta de Hierro, 28040 Madrid, Spain; avela@ucm.es (A.I.V.A.); garayzab@ucm.es (J.F.F.-G.); 3 Azienda Sanitaria Locale di Biella, via Don Sturzo 20, 13900 Biella, Italy; raffaella.barbero@aslbi.piemonte.it

**Keywords:** aquaculture, fish diseases, outbreak, brook trout, *Lactococcus garvieae*

## Abstract

**Simple Summary:**

*Lactococcus garvieae* is the etiological agent of lactococcosis, a bacterial disease affecting many species of fish and causing major economic losses in aquaculture. In this study we described, for the first time, the isolation of *L. garvieae* in brook trout farmed in northwestern Italy by performing a molecular and epidemiological characterization. Results confirmed water as vehicle of infection, favoring the transmission of the pathogen between rainbow trout farmed in the upstream compartments of a raceways system and the brook trout located in downstream tanks.

**Abstract:**

Lactococcosis is a fish disease of major concern in Mediterranean countries caused by *Lactococcus garvieae*. The most susceptible species is the rainbow trout (*Oncorhynchus mykiss*), suffering acute disease associated with elevated mortalities compared to other fish species. References reported that other salmonids are also susceptible to the disease, but no mortality outbreak has been described to date. The aim of this study was to present a mortality outbreak that occurred in brook trout (*Salvelinus fontinalis*) farmed in northwestern Italy during the summer of 2018. Fish exhibited clinical signs, such as exophthalmos, diffused hemorrhages localized in the ocular zone, hemorrhagic enteritis, and enlarged spleen. *L. garvieae* was isolated in all fish. Molecular and epidemiological characterization of the isolates, through Pulsed Field Gel Electrophoresis (PFGE), confirmed the initial hypothesis of water as vehicle of infection favoring transmission between rainbow trout farmed in upstream compartments and brook trout located in downstream tanks. Moreover, several environmental conditions affected and promoted the outbreak, among them the high-water temperature, which probably induced a physiological stress in brook trout, being way above the optimal temperature for this species, increasing the susceptibility to infection.

## 1. Introduction

Streptococcosis is a disease responsible for septicemic processes in several freshwater and marine fish [1]. From an etiological point of view, streptococcosis are strictly linked to water temperature and are considered seasonal diseases divided into two groups: warm water infections that affect fish at a water temperature above 15 °C and cold-water infections pathogenic only for fish at temperatures below 15 °C [2]. During the last decade, thanks to the development of new techniques of diagnosis based on genotypic characteristics, numerous changes in the taxonomy of bacteria involved in streptococcosis have been made, with the description of five bacterial genera: *Streptococcus*, *Enterococcus, Lactococcus*, *Vagococcus*, and *Carnobacterium* [3,4,5]. Etiological agents of warm water streptococcosis are represented by four species: *Lactococcus garvieae* [6], *Streptococcus iniae* [7], *Streptococcus agalactiae* [8], and *Streptococcus parauberis* [9].

Lactococcosis is caused by *L. garvieae*, a Gram-positive coccus, isolated from various species of aquatic animals [10,11,12] and from mastitis in cows and buffalos [10,13]. This microorganism has also been isolated from several clinical cases in humans, suggesting that *L. garvieae* should be considered as potential zoonotic agent [14]. Lactococosis is a disease of major concern in several trout farms mostly located on the plain causing high economic losses that can exceed approximately 50–80% of the total production [2,6,15]. The disease was described for the first time in Japan in an intensive farm of rainbow trout (*Oncorhynchus mykiss*) [16]. In regards to Europe, the first isolation was reported in Spain in 1993 [17] and, one year later, the same pathogen was also detected in Italy in intensive rainbow trout farms located in North Italy [6,15]. From that point on, this pathogen rapidly spread throughout the southern part of the European continent [18] thanks to its high virulence, the lack of suitable control methods, and the movement of infected fish [19].

Published data confirm a global increase of outbreaks affecting rainbow trout in several countries all over the world, such as Australia, South Africa, Japan, Taiwan [5], and the USA [20]. Therefore, *L. garvieae* can be considered a cosmopolitan pathogen. Transmission of the disease occurs by horizontal mechanisms, mainly through water, fish injuries, and by the fecal–oral route [21]. *L. garvieae* is responsible of a hyperacute and hemorrhagic septicemia, although the evolution of the disease strictly depends on environmental conditions, such as water temperature and water microbiological quality [18].

The gross pathology consists in a rapid and general anorexia, melanosis, lethargy, loss of orientation, and erratic swimming. Typical external signs of affected fish are exophthalmos, the presence of hemorrhages in the periorbital and intraocular area at the base of fins, in the perianal region, and in the buccal region. It is also very common to observe fish with swollen abdomens and anal prolapses. Algöet and co-workers [22] reported that other salmonids such as Atlantic salmon (*Salmo salar*), brown trout (*Salmo trutta*), and brook trout (*Salvelinus fontinalis*) were also susceptible to the disease, but no mortality outbreaks have been described in brook trout to date.

Italy is the first producer of freshwater salmonids in the EU and more than 65% of the production is yielded in North Italy. Rainbow trout is the most important farmed fish species but, even if in a smaller scale, other salmonids such as brook trout are produced, being sold for human consumption in both fresh and smoked form.

The aim of this study was to describe the first isolation of *L. garvieae* in brook trout farmed in northwestern Italy and to perform a molecular and epidemiological characterization of the isolates.

## 2. Material and Methods

A septicemic outbreak with typical symptoms of lactococcosis occurred between July and August 2018 in a trout farm located in northwestern Italy. The raceway system contained rainbow trout (300 q; 300 g medium sized) in the upstream compartments, and brook trout (150 q; 250–300 g medium sized) in the downstream tanks. Ninety-five percent of both rainbow trout and brook trout exhibited clinical signs, with a mortality rate of 70% (8–10 kg/day fish losses) and 75% (8–10 kg/day fish losses), respectively. The water temperature was 18–19 °C, at the limit of fish tolerance.

On 8 August 2018, 25 recently moribund brook trout (250–300 g weight) and 25 rainbow trout (300 g weight) were sent refrigerated within 3 h to the Fish Diseases Laboratory of the Instituto Zooprofilattico Sperimentale del Piemonte, Liguria e Valle d’Aosta for further microbiological analysis. Fish were necropsied, and samples of kidney and brain were aseptically removed and used for further microbiological analyses.

For microbiological analysis, clinical specimens were grown on Columbia blood agar plates (Liofilchem, Italy) and incubated at 22 °C ± 2 °C for 72 h. Bacterial isolates were initially identified by using commercial Rapid ID32 STREP strips (bioMérieux, France) after an incubation period of 24 h at 37 °C ± 2 °C. Additional phenotypic identification was carried out by the VITEK MS system (bioMérieux, France) according to the manufacturer’s instructions. Phenotypic identification of *L. garvieae* isolates was confirmed by a species-specific PCR [23].

*L. garvieae* isolates (n = 16) were molecularly characterized (10 isolates from rainbow trout and six isolates from brook trout) by Pulsed Field Gel Electrophoresis (PFGE) with the enzymes *Apa*I and *Sma*I (MBI Fermentas) as described by Vela and co-workers [24] with the following modifications: running time, 21 h; temperature, 14 °C; voltage gradient, 6 V/cm; and included angle, 120 °C, with an initial pulse time of 0.1 s and a final pulse time of 25 s. *Xba*I-digested DNA from *Salmonella enterica* serotype Braenderup H9812 was used for molecular weight size determination.

## 3. Results and Discussion

**Figure 1:**
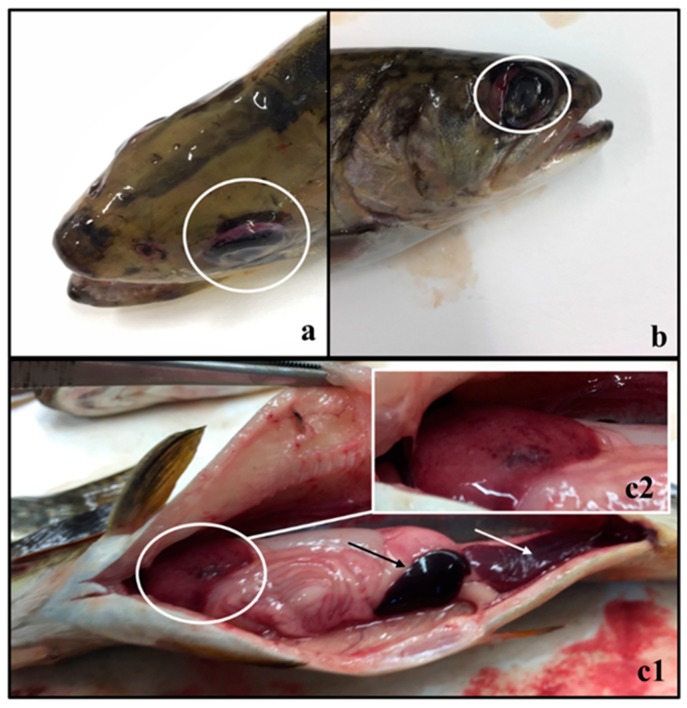
Clinical signs of lactococcosis observed in brook trout: (**a**) exophthalmos and haemorrhages in the periorbital and intraocular area; (**b**) ruptured globe; (**c1**) enlarged spleen (black arrow), hemorrhagic enteritis (white arrow), and (**c2**) diffused haemorrhages on liver.

Both trout species showed typical signs of lactococcosis, with erratic swimming, anorexia, lethargic behavior, melanosis (only in rainbow trout), exophthalmos, and hemorrhages in the periorbital and intraocular cavity as the most significant clinical signs and pathological findings being observed. Clinical signs (Figure 1) included diffuse hemorrhagic areas on the surface of internal organs (especially in liver and swim bladder) as well as in the ocular zone (with cases of ruptured globe), buccal area, opercula, fins and perianal area, hemorrhagic enteritis, enlarged spleen, and anal prolapse.

Pure culture of gram-positive, catalase-negative cocci were isolated in Columbia blood agar from the kidney and brain samples of all rainbow trout and brook trout analyzed. The morphological characteristics of the colonies and microscopic examination by Gram staining were identical for all the clinical isolates grown from the kidney and brain samples. Only 16 isolates were further biochemically identified and genetically characterized. Bacterial isolates were accurately identified as *L. garvieae* with both the Rapid ID32 STREP (API code: 30323500030, ID 99.9%) and VITEK MS systems (ID 99.9%). Additionally, all isolates gave the expected 1100 bp PCR amplification product, which is specific for *L. garvieae* [23] confirming the biochemical identification.

**Figure 2:**
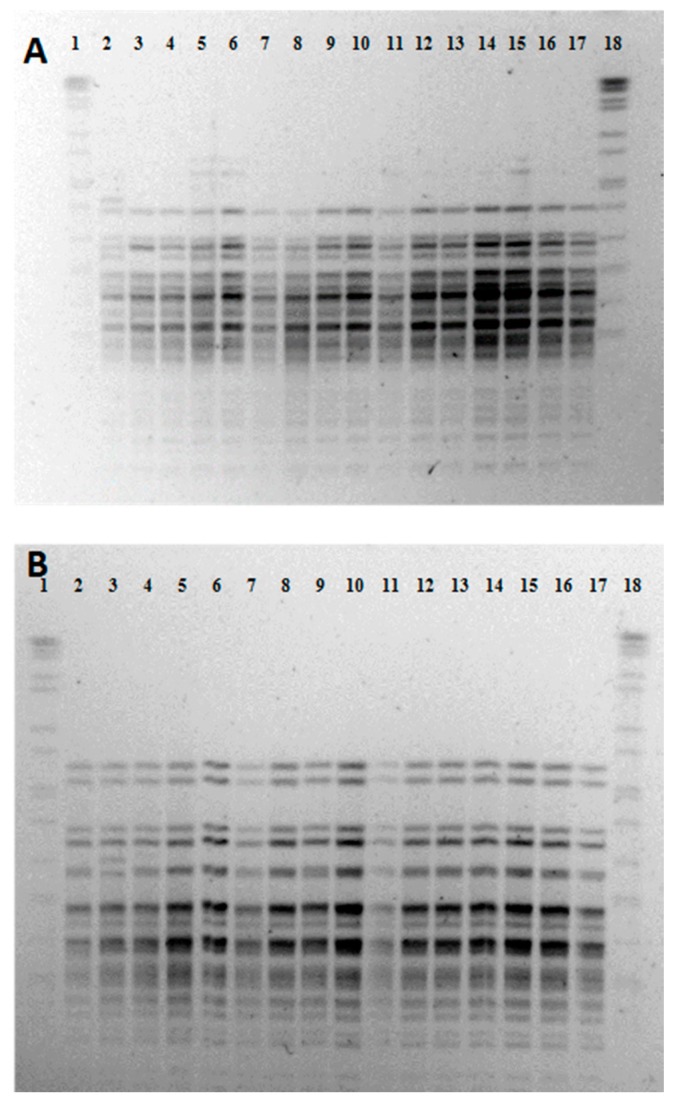
Pulsed-field gel electrophoresis patterns of *Ap*aI (**A**) and *Sma*I (**B**) digests of genomic DNA of *Lactococcus garvieae* clinical isolates. Lane 1 and 18, *Salmonella* serotype Branderup strain H9812; lanes 2–6 and lane 13, brook trout isolates from samples S-8, S-9, S-10, S-11 S-12, and S-7; lanes 7–12 and lanes 14–17, rainbow trout isolates of samples T-1, T-2, T-3, T-4, T-5, T-6, T-7, T-13, T-14, and T-15.

After Pulsed Field Gel Electrophoresis (PFGE) analysis, the 10 *L. garvieae* isolated from rainbow trout exhibited undistinguishable PFGE patterns with each of the enzymes (Figure 2A,B, lanes 7 to 12 and lanes 14 to 17), which suggests that they represent a single clone of *L. garvieae* responsible for the outbreak in this trout species. The PFGE pattern obtained with the *Apa*I enzyme was undistinguishable from those previously identified in Italy [24]. This result suggests the potential of *L. garvieae* for long term persistence in the aquatic environment and/or on asymptomatic infected fish, being responsible for different lactococcosis outbreaks overtime.

Clinical isolates of *L. garvieae* from brook trout exhibited three and two PFGE patterns using *Apa*I (Figure 2A, pulsotype 1, lane 2; pulsotype 2, lane 3; and pulsotype 3, lanes 4–6 and 13) and *Sma*I (Figure 2B, pulsotype 1, lanes 2, 4–6, and 13 and pulsotype 2, lane 3) enzymes, respectively. Four (lanes 4–6 and 13) out of the six *L. garvieae* brook trout isolates characterized by PFGE, with the *Apa*I enzyme, exhibited a pulsotype undistinguishable from that found in rainbow trout isolates (Figure 2A), while the other two isolates exhibited different, although closely related, PFGE patterns (Figure 2A, lanes 2 and 3).

This result indicates that different strains of *L. garvieae* can be found in a lactococcosis outbreak. *L. garvieae* is a genetically heterogeneous microorganism [25]. However, 14 out of the 16 isolates of *L. garvieae* of both trout species characterized by PFGE (87.5%) exhibited the same pulsotype with the enzyme *Apa*I. This fact, together with their isolation in pure culture from internal organs of different animals over a period of two months, is indicative of its clinical significance. *L. garvieae* is able to infect many different fish species, such as yellowtail, tilapia, Japanese eel, olive flounder, grey mullet, catfish, wild wrasse, black rockfish, amberjack, kingfish, or giant fresh water prawn [7,26,27,28,29,30,31,32]. This is the first report of a lactococcosis outbreak affecting brook trout, which expand the range of fish species that can be affected by this pathogen.

There are several aquatic environmental factors such as fish stress, overcrowding, mishandling, poor water quality, and principally water temperature that influence the appearance of lactococcosis outbreaks [5]. Epidemiological data indicates that lactococcosis outbreaks usually occur during summer when water temperatures rise over 14–15 °C [17,33]. Therefore, the high-water temperature (18–19 °C) of the river was the most likely predisposing factor that contributed to the development of the outbreak in both rainbow and brook trout. Moreover, the temperature of the water was in the upper limit of the thermal tolerance for brook trout, which could induce cellular and endocrine stress responses [34] that might have increased susceptibility to *L. garvieae* infection in this fish species. No deficiencies in management practices were identified in either of both affected fish farms. Other factors related with the water quality, such as pH, oxygen, or ammonium concentrations, were not recorded and are therefore impossible to be evaluated for their influence in the appearance and severity of the lactococcosis outbreak. Furthermore, no control measures (vaccination or therapeutic treatment) were used.

The application of chemotherapeutic agents is an unsustainable strategy in the control of lactococcosis due to the development of antibiotic resistance [12].

The application of preventatives in terms of biosecurity measures, such as destruction of moribund fish, regular and appropriate disinfection of equipment, improvement of health management measures, and immunization of healthy fish, are highly recommended in trout farms, particularly in those that use rivers as a source of water [35,36,37,38]. In fact, *L. garvieae* reach aquatic environments through contaminated feces of infected or diseased fish [39] being further transmitted through the fecal–oral route [21]. The tanks with brook trout were located downstream to those with rainbow trout. Therefore, it is likely that dissemination of *L. garvieae* from the rainbow trout to brook trout occurred through the contamination of the water.
